# Chronic exposure to a synthetic cannabinoid alters cerebral brain metabolism and causes long-lasting behavioral deficits in adult mice

**DOI:** 10.1007/s00702-023-02607-8

**Published:** 2023-02-28

**Authors:** Caroline Bouter, Frederik Wilhelm Ott, Daniel Günther, Lukas Weig, Fabian Schmitz-Peiffer, Mahriban Rozyyeva, Nicola Beindorff, Yvonne Bouter

**Affiliations:** 1 Department of Psychiatry and Psychotherapy, University Medical Center, Georg-August-University, Göttingen, Germany; 2 Department of Nuclear Medicine, University Medical Center Göttingen, Göttingen, Germany; 3 Berlin Experimental Radionuclide Imaging Center (BERIC), Charité - Universitätsmedizin Berlin, Berlin, Germany; 4 Nuclear Medicine, Charité - Universitätsmedizin Berlin, Berlin, Germany

**Keywords:** Cannabis, Medical marijuana, FDG-PET, WIN 55,212-2, Anxiety, Behavior testing

## Abstract

In recent years, there has been growing evidence that cannabinoids have promising medicinal and pharmacological effects. However, the growing interest in medical cannabis highlights the need to better understand brain alterations linking phytocannabinoids or synthetic cannabinoids to clinical and behavioral phenotypes. Therefore, the aim of this study was to investigate the effects of long-term WIN 55,212-2 treatment—with and without prolonged abstinence—on cerebral metabolism and memory function in healthy wildtype mice. Adult C57BI/6J mice were divided into two treatment groups to study the acute effects of WIN 55,212-2 treatment as well the effects of WIN 55,212-2 treatment after an extended washout phase. We could demonstrate that 3 mg/kg WIN 55,212-2 treatment in early adulthood leads to a hypometabolism in several brain regions including the hippocampus, cerebellum, amygdala and midbrain, even after prolonged abstinence. Furthermore, prolonged acute WIN 55,212-2 treatment in 6-months-old mice reduced the glucose metabolism in the hippocampus and midbrain. In addition, Win 55,212-2 treatment during adulthood lead to spatial memory and recognition memory deficits without affecting anxiety behavior. Overall we could demonstrate that treatment with the synthetic CB1/CB2 receptor aganist Win 55,212-2 during adulthood causes persistent memory deficits, especially when mice were treated in early adulthood. Our findings highlight the risks of prolonged WIN 55,212-2 use and provide new insights into the mechanisms underlying the effects of chronic cannabinoid exposure on the brain and behavior.

## Introduction

Cannabis is one of the most commonly used illicit drugs and the use of cannabis has increased over the last decades (Ehrenreich et al. 1999; Raphael et al. 2005; Nations 2018; Hall et al. 2016). In parallel, there is a worldwide trend towards decriminalization and legalization of cannabis. Several countries around the world have already legalized the recreational use of cannabis, including Uruguay, Australia, Canada and some states in the United States (Hammond et al. 2020; Burnett et al. 2022).

The endocannabinoid system (ECS) is a complex, highly conserved endogenous regulatory system that is involved in many physiological functions including brain development, cognitive and memory functions, inflammatory processes as well as respiratory, cardiovascular, and reproductive processes (Salzet et al. 2000; Pagano et al. 2022). Alterations in components of the ECS have been shown to play a role in various pathological conditions such as cancer, cardiovascular, and neurodegenerative diseases (Lowe et al. 2021). Therefore, pharmacological modulation of the system has gained significant interest in medical research with the goal of developing drugs capable of affecting signaling pathways downstream of the ECS.

Natural and synthetic cannabinoids possess anti-inflammatory, anti-proliferative, and anti-analgesic properties (Fouda et al. 2022; Kis et al. 2019; McDougall and McKenna 2022; Nagarkatti et al. 2009). Therefore, there has been growing interest in cannabis as a potential therapeutic agent (Henderson et al. 2021; Haddad et al. 2022; Khalsa et al. 2022; Laws and Smid 2022; Pagano et al. 2022; Bergamaschi et al. 2011). For example, modulation of the endocannabinoid system seems to have beneficial effects on neurodegenerative diseases, particularly Alzheimer’s disease (Wenger et al. 2021; Cooray et al. 2020b; Schmole et al. 2018; Abd-Nikfarjam et al. 2023; Janefjord et al. 2014). Thus, activation of cannabinoid receptors 1 and 2 (CB1 and CB2) has been shown to have neuroprotective effects, reducing beta-amyloid, tau, and neuroinflammation in vitro and in vivo in rodents (Aso et al. 2012; Chen et al. 2011; Janefjord et al. 2014; Esposito et al. 2007; Cooray et al. 2020a). The synthetic CB1 and CB2 agonist WIN 55,212-2 has previously been shown to modulate neuroinflammation, increase beta-amyloid clearance, and improve cognitive impairment in several preclinical studies using cell culture and rodent models of Alzheimer’s disease (Velikova et al. 2020; Sheng et al. 2005; Martin-Moreno et al. 2012; Jin et al. 2004; Jiang et al. 2005). However, to take full advantage of the beneficial effects of cannabinoids like WIN 55,212-2 for the treatment of diseases in the sense of ‘drug re-purposing’, more data on potential side effects of prolonged treatment are needed.

Regular cannabis use has been associated with a variety of short- and long-term adverse effects including an increased risk of developing psychiatric symptoms like acute psychosis, paranoia, hallucinations, and mania (Helle et al. 2016; Rodrigo and Rajapakse 2009; Hindley et al. 2020). In rodents chronic treatment with WIN 55,212-2 in adolescence resulted in long-term impairments in object and social recognition, sensory-motor gating, social behavior and social play, as well as self-care (Schneider and Koch 2003; Schneider et al. 2008; Acheson et al. 2011).

However, the underlying brain alterations linking cannabinoids to clinical and behavioral phenotypes remain debated. Therefore, neuroimaging can be a powerful tool to non-invasively investigate the effects of cannabis exposure on brain function in both humans and animal models. Positron emission tomography (PET) with ^18^F-fluorodeoxyglucose (FDG) can be used to evaluate brain activity and therefore provide important insights in the effects of cannabinoids like WIN 55,212-2 on the brain.

Given the projected increase in cannabis use and the growing interest in the therapeutic potential of cannabinoids, it is critical to understand the effects of cannabis constituents and synthetic cannabinoids on the brain and behavior. Therefore, the aim of this study was to investigate the effects of long-term WIN 55,212-2 treatment, with and without prolonged abstinence, on cerebral metabolism and memory function in healthy adult wildtype mice.

## Material and methods

### Animals and drug treatment

C57BL/6J wildtype (WT) mice (Jackson Laboratories, Bar Harbor, ME, USA) were used in this study with an equal distribution of female and male mice. Mice were housed in individually ventilated cages (IVC, 32 × 16 × 14 cm; Tecniplast, Hohenpeißenberg, Germany) in a controlled environment on a 12/12 h light/dark cycle in groups randomly divided up to five. Water and food were available ad libitum. All animals were handled according to the German guidelines and EU legislation for animal care and the experiments were approved by the local authorities (Niedersächsisches Landesamt für Verbraucherschutz [16/2364, 17/2614] and Landesamt für Gesundheit und Soziales LAGeSo Darwinstr. 15, 10,589 Berlin [65/18, 260/19]).

**Fig. 1:**
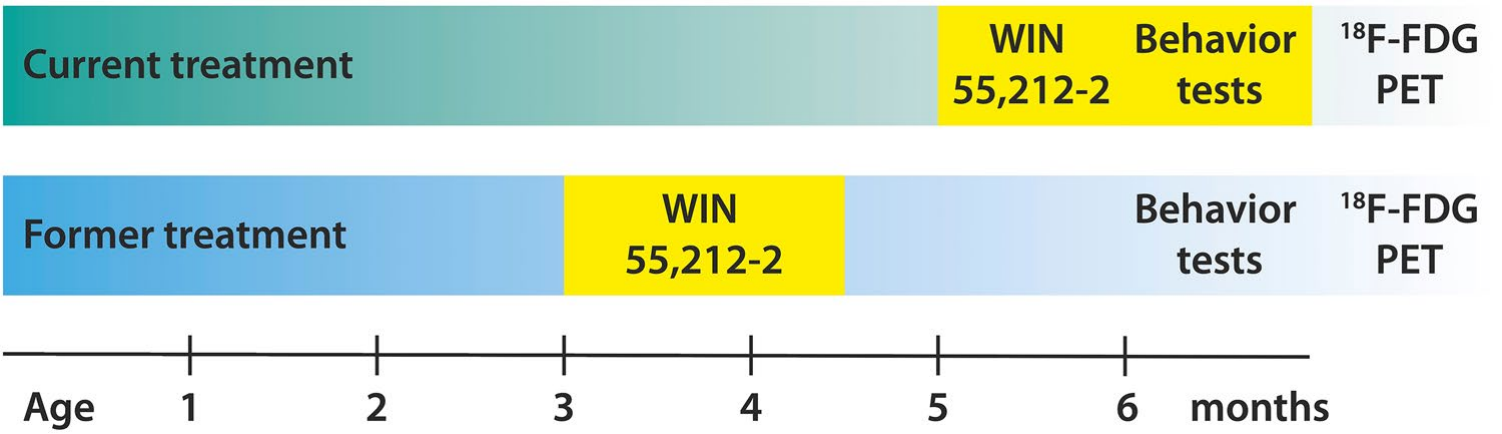
Schematic overview of the experimental design. Mice were treated with WIN 55,212-2 or a vehicle solution over 42 consecutive days starting at 3 (former) and 5 months (current), respectively. Behavior tests started at 6 months and ^18^FDG-PET was performed at 6.5 months

The CB1/CB2 receptor agonist (R)-( +)-[2,3-dihydro-5-methyl-3-(4-morpholinyl-methyl) pyrrolo-[1,2,3-d,e]-1,4-benzoxazin-6-yl]-1-naphthalenyl-methanone (WIN 55,212-12, Sigma-Aldrich, St. Louis, MO, USA) was dissolved in a vehicle solution consisting of 5% Tween 80 (Carl Roth GmbH, Karlsruhe, Germany), 5% Ethanol (100%), and 90% NaCl (0.9%). Treatment started at 3 months (in the following called ‘former’) or 5 months (in the following called ‘current’), respectively, and lasted for six weeks (Fig. 1). Mice were assigned to either WIN 55,212-2 or vehicle treated groups and treated daily with an intraperitoneal injection volume of 10 ml/kg. The drug was administered at a dosage of 3 mg/kg. Behavioral testing started for all mice at 6 months.

### Behavior testing

To detect possible cognitive and behavioral alterations caused by WIN 55,212-2 treatment, animals were tested in a battery of anxiety and memory tests. All mice were analyzed at the age of 6 months and testing lasted 15 days. Mice were kept on a 12 h/12 h inverted light cycle and all behavior experiments were performed during the dark phase between 7 a.m. and 7 p.m.

### Open field

The open field (OF) test was used to analyze explorative behavior and spontaneous motor activity (Himanshu et al. 2020). Mice were tested for 5 min in a 50 × 50 cm arena with 38 cm high walls. ANY-Maze video tracking software (Stoelting Co, Wood Dale, IL, USA) was used to record the distance traveled and the time spent in the center area. Between mice the maze was cleaned with 70% ethanol to diminish odor cues.

### Elevated plus maze

The elevated plus maze (EPM) was used to assess anxiety-related and exploratory behavior (Himanshu et al. 2020; Kraeuter et al. 2019). The apparatus was made of four arms (5 cm width × 15 cm length) extending at 90° angles from a central area (5 cm width × 5 cm length) raised 75 cm above a padded surface (Schleicher et al. 2022).

The maze consisted of two closed arms facing each other, surrounded on three sides by a 15 cm high transparent plastic wall, and two open arms. Mice were placed in the center facing an open arm and were allowed to explore the maze freely for 5 min. Distance traveled and the time spent in each arm were recorded using the ANY-Maze tracking software (Stoelting Co, Wood Dale, IL, USA). Anxiety-like behavior was calculated based on the time spent in the open arms, with longer times spent in the open arms corresponding to lower levels of anxiety (Schleicher et al. 2022). After each mouse the EPM was cleaned using 70% ethanol to eliminate odor cues.

### Dark light box

The Dark Light box (DLB) was used to assess anxiolytic-like or anxiogenic-like activity in mice (Bourin and Hascoet 2003). The test is based on the innate light aversion and the spontaneous exploratory behavior of rodents (Himanshu et al. 2020).

The test was performed in a grey plastic arena (73 cm × 25 cm × 32 cm) divided into two areas: a smaller dark area (31 cm × 25 cm) and a larger light area (42 cm × 31 cm). Mice were able to freely move from one compartment to the other through a small opening (5 cm × 5 cm) in the wall. Each mouse was introduced into the light area facing the wall and was allowed to explore the space freely for 5 min. ANY-Maze video tracking software (Stoelting Co., Wood Dale, IL, USA) was used to record the time spent in each area, the latency too enter the dark area and the number of crossings.

### Novel object recognition test

In order to assess recognition memory and novelty preference (Antunes and Biala 2012), the novel object recognition test (NOR) was performed in an open-field arena made of gray plastic (50 × 50 cm) as previously described (Schleicher et al. 2022). Twenty-four hours after the Open field test, NOR was performed in the same arena containing two identical objects. Mice were allowed to freely explore the box for 5 min. The following day, mice were placed back in the same arena with one familiar and one novel object. The animals were tracked using an automated video tracking system (ANY-maze, Stoelting Co, USA) and the time spent exploring each object as well as the distance traveled was recorded. The objects and the box were cleaned with 70% ethanol between each mouse to remove any lingering scents.

The percentage of exploration time for the novel object was calculated as follows:

$$\text{Novel\,Object }\left[\text{\%}\right]= \left(\frac{\text{Time\,(Novel\,Object)}}{\text{Time\, (Novel\,Object)}+\text{Time\,(Familiar\,Object)}}\times 100\right)$$

### Morris water maze

In order to assess spatial reference memory, the Morris water maze test was used as described before (Bouter et al. 2018; Schleicher et al. 2022). Briefly, the test relies on spatial cues to locate a submerged hidden platform (10 cm diameter) in a circular pool (110 cm diameter) filled with non-transparent tap water. The pool was divided into four virtual quadrants that were defined based on their spatial relationship to the platform: left (L), right (R), opposite (O) and target (T) quadrant, which contains the goal platform (Curdt et al. 2022).

Mice were first subjected to 3 days of cued training to test if mice have the ability to swim and an intact vision. During the cued training the platform was marked with a triangular flag and mice were given 60 s to find the submerged platform. The location and the starting point changed between the trials. Mice received 4 cued training trials per day with an average inter-trial interval of 15 min.

Twenty-four hours after the last day of cued training, mice performed 5 days of acquisition training (4 trials/day). During the acquisition training proximal visual cues were attached to the outside of the pool and the flag was removed from the platform. The platform location remained stationary throughout training and mice were introduced into the pool from one of four predefined entry points (Schleicher et al. 2022).

The platform was removed from the pool at the final probe trial and mice swam freely for one minute assessing their swimming path and swimming speed. ANY-Maze video tracking software (Stoelting Co.,Wood Dale, USA) was used to record swimming speed, escape latency, and quadrant preference.

**Fig. 2:**
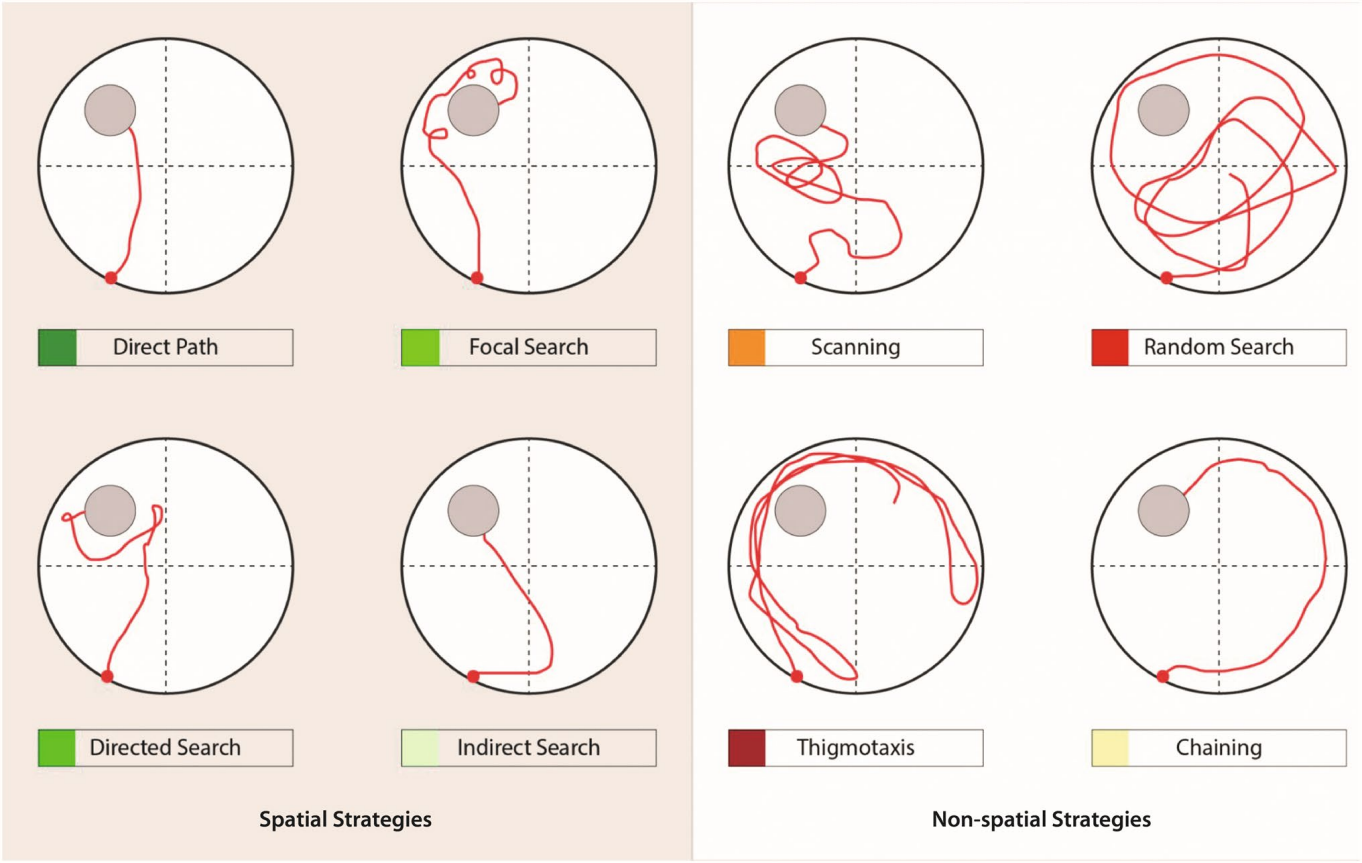
Representative examples of possible search strategies in the Morris Water Maze. ‘Direct path’, ‘directed search’, ‘focal search’ and ‘indirect search’ are considered as spatial strategies. Non-spatial strategies include ‘scanning’, ‘thigmotaxis’, ‘random search’ and ‘chaining’

In addition, searching strategies were analyzed during the acquisition training and probe trial with Pathfinder (Jason Snyder Lab, Vancouver, Canada) (Curdt et al. 2022; Cooke et al. 2019). Spatial strategies included ‘direct path’, ‘directed search’, ‘focal search’ and ‘indirect search’ (Fig. 2). ‘Chaining’, ‘scanning’, ‘random search’ and ‘thigmotaxis’ were considered as non-spatial strategies. Pathfinder categorizes each trial according to 1 of 8 possible strategies. ‘direct path’ (Ideal path error [IPE] ≤ 1250 mm; Heading error ≤ 40°), ‘directed search’ (time in angular corridor ≤ 70% of trial; distance covered ≤ 4000 mm; IPE ≤ 15,000 mm), ‘focal search’ (distance to swim path centroid ≤ 30% of radius; distance to goal ≤ 30% of radius; distance covered ≥ 1000 mm and ≤ 4000 mm), ‘indirect search’ (IPE ≤ 3000 mm; average heading error ≤ 360°), ‘chaining’ (time in annulus zone ≥ 90% of trial; quadrants visited ≥ 4; area of maze traversed ≤ 40% of maze), ‘scanning’ (area of maze traversed ≥ 5% and ≤ 20% of maze; average distance to maze center ≤ 60% of radius), ‘random search’ (area of maze traversed ≥ 10% of maze) and ‘thigmotaxis’ (time in full thigmotaxis zone ≥ 65% of trial; time in smaller thigmotaxis zone ≥ 35% of trial; total distance covered ≥ 4000 mm). The different spatial parameters were adjusted to the experimental setup (goal position [x/y]: 275, 775; goal diameter: 200; maze diameter: 1100; maze center [x/y]: 550, 550; angular corridor width: 40; chaining annulus width: 200; thigmotaxis zone size: 50) (Curdt et al. 2022).

### ^18^F-FDG-PET/CT

7-month-old former (n = 8) and current (n = 5) WIN-treated WT mice and untreated age-and sex-matched WT mice (n = 5) were scanned with ^18^F-FDG-PET/CT using a small animal nanoScan PET/CT (mouse whole body volume coil, diameter 35 mm, Mediso, Hungary) as previously described (11, 16). Following an overnight fasting period, blood glucose levels were measured before tracer injection in each mouse. 15.57–25.54 MBq ^18^F-FDG (Mean 21.71 MBq) were administered into a tail vein with a maximum volume of 200 μl followed by an uptake period of 45 min in which mice were awake and kept warm. After the uptake period, mice were anesthetized with 1–2% isoflurane supplemented with oxygen (0.5 l/min, cp-pharma, Burgdorf, Germany). PET acquisition time was 20 min and scans were performed in a single mouse model. Mice were kept on a heated bed (37 °C) during the scans and the respiratory rate was measured. CT-based attenuation correction was conducted.

### Image analysis

PMOD v3.9 (PMOD Technologies, Switzerland) was used for analysis of PET images were analyzed using as previously described (Bouter et al. 2018).

An MRI-based mouse atlas template was used to define several volumes of interest (VOI) within the brain including amygdala, brain stem, cerebellum, cortex, hippocampus, hypothalamus, midbrain, olfactory bulb, septum/basal forebrain, striatum, and thalamus. Volumes of the regions were between 10 mm^3^ (amygdala) and 0.3 cm^3^ (cortex) in size. The template was placed on each individual mouse brain CT and images were fused with the PET images of each mouse. VOI statistics (kBq/cc) were generated for all VOI and standardized uptake values (SUV) were calculated [SUV = tissue activity concentration average (kBq/cc) × body weight (g)/ injected dose (kBq)]. SUV were further corrected for blood glucose levels [Glc = SUV × blood glucose level (mg/dl)].

### Statistical analysis

All statistics were performed usind GraphPad Prism Version 9 (GraphPad Software, San Diego, CA, USA). Differences between groups were tested using unpaired t-test, one-way analysis of variance (ANOVA) followed by Bonferroni multiple comparison or two-way analysis of variance (ANOVA) followed by Bonferroni multiple comparisons as indicated. Data are shown as mean + / − standard deviation. Significance levels are given as follows: *p < 0.05; **p < 0.01; ***p < 0.0001.

## Results

### Prolonged WIN 55,212-2 treatment impairs recognition memory

**Fig. 3:**
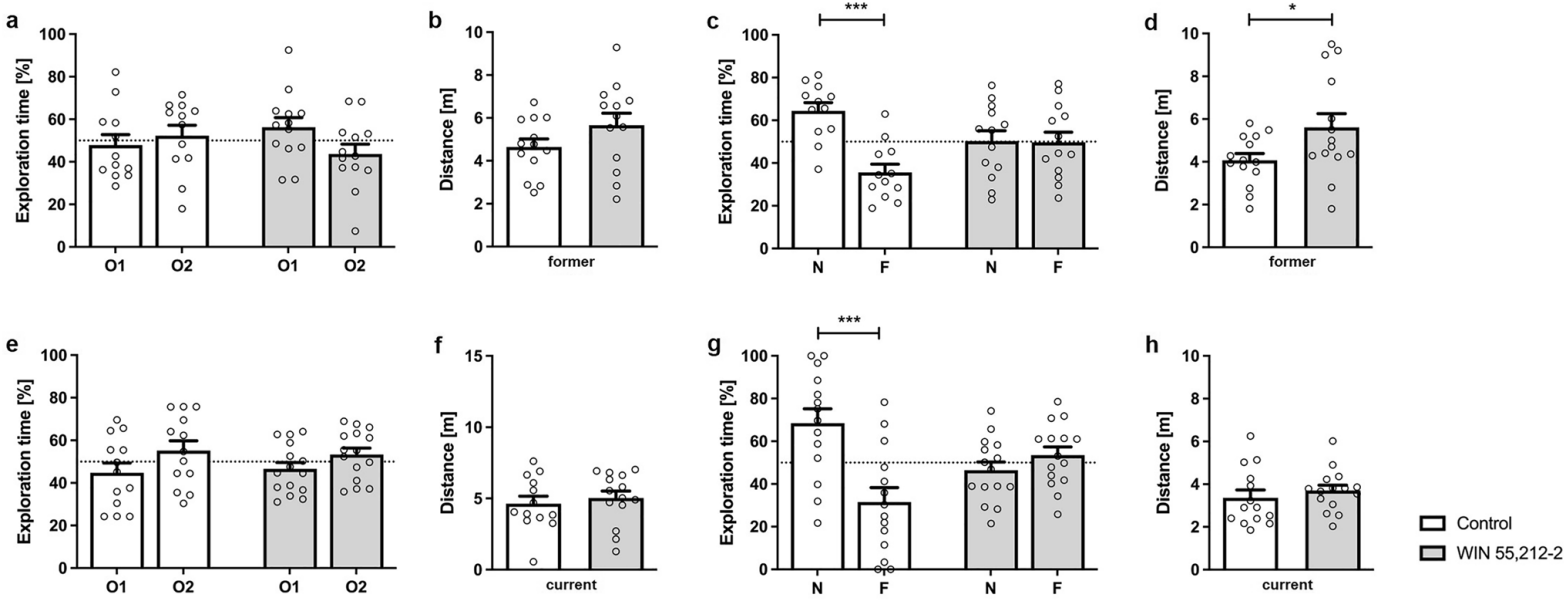
WIN 55,212-2-treatment impairs recognition memory of C57BL/6 J in the novel object task. **a**, **e** During the training phase, all mice, independent of the treatment, spent equal amounts of time with two similar objects (O1, O2). During the testing phase, only vehicle-treated mice of the former (**c**) and vehicle mice of the current group (**g**) showed a significant preference for the novel object (N). In contrast, WIN-treated mice, regardless of treatment start, did not discriminate between the novel (N) and the familiar object (F). Distance traveled during the training phase (**b**) did not differ between WIN-treated and control mice. In contrast, **d** former WIN-treated mice traveled significantly more than control animals. Distance traveled did not differ between current WIN-treated mice and control animals during the training day (**f**) or testing phase (**h**). Fifty percent chance level is indicated by a dashed line. Paired t-test (**a**, **c**, **e**, **g**) and unpaired t-test (**b**, **d**, **f**, **h**); n = 14–15. Data presented as mean ± S.E.M; *p < 0.05, ***p < 0.01

Recognition memory was assessed using the NOR. Former WIN-treated WT mice showed impaired recognition memory as they were unable to distinguish between a new (N) and familiar (F) object. During the exploration phase on the first day, WIN-treated and control mice spent equal amounts of time exploring the objects (Fig. 3a; one-way repeated measures ANOVA, treatment former: F(3,46) = 1.3, p = 0.2677). In addition, distance traveled did not differ between treatment groups on the training day (Fig. 3b; unpaired t-test, former: F(13,13) = 2.272, p = 0.1694). When tested for recognition memory 24 h later, control mice showed a significant preference towards the novel object (Fig. 3c; one-way repeated measures ANOVA, *treatment* former: F(3,46) = 7.048, p < 0.001; Bonferroni comparisons: vehicle *N* vs* F*: p < 0.001). In contrast, WIN-treated mice spent an equal amount of time exploring the familiar and novel object, indicating that they were unable to discriminate between the two objects (Bonferroni comparisons: WIN *N* vs* F*: p > 0.05). During the testing day WIN-treated mice traveled significantly more than control animals (Fig. 3d; unpaired t-test, former: F(13,13) = 4.073, p = 0.0407).

Similarly, mice that were currently treated with WIN showed an impaired recognition memory as they did not discriminate between the novel and familiar object on the testing day (Fig. 3g, two-way repeated measures ANOVA, *treatment* current: F(3,52) = 7.8, p = 0.0002; Bonferroni comparisons: WIN *N* vs* F*: p > 0.05). In contrast, control WT mice spent significantly more time exploring the novel object (Bonferroni comparisons: vehicle *N* vs* F*: p < 0.001). Both groups spend a similar amount of time with the objects during the exploration phase on the first day (Fig. 3e; one-way repeated measures ANOVA, *treatment*: F(3,52) = 1.7, p = 0.1682). The distance traveled did not differ between current vehicle and WIN-treated mice (Fig. 3f, h; unpaired t-test, current: day1 F(13,14) = 1.072, p = 0.5859; day 2*:* F(13,14) = 1.886, p = 0.4444).

### Prolonged WIN 55,212-2 treatment impairs spatial memory

In the cued training, all former and current treated mice showed a significant decline in escape latency over time with no significant difference between the groups (data not shown, two-way repeated measures ANOVA, treatment former: F(1,24) = 0.04519, p = 0.8335; treatment current: F(1,28) = 3.829, p = 0.0612). However, former treated WIN-mice swam significantly faster than control mice (data not shown, two-way repeated measures ANOVA, treatment former: F(1,24) = 11.62, p = 0.0023). No overall differences in swimming speed could be detected between current WIN-treated mice and controls (data not shown; two-way repeated measures ANOVA, treatment current: F(1,28) = 1.006, p = 0.3245). The cued training period revealed that all mice had an intact vision and the motoric abilities to swim.

**Fig. 4:**
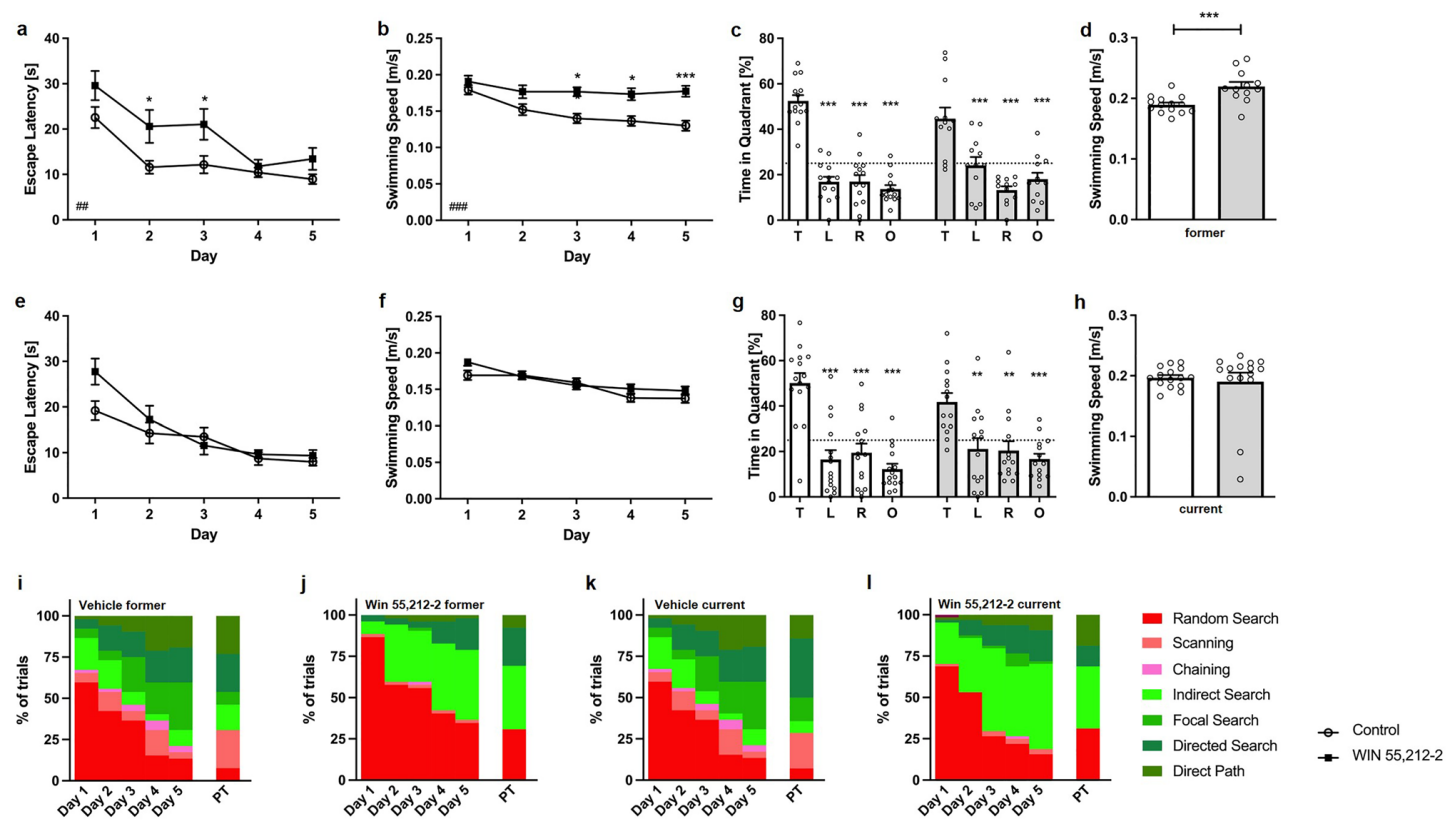
Effects of prolonged WIN 55,212-2-treatment on spatial learning and spatial reference memory in the Morris water maze. **a** Former WIN-treated mice required significantly more time to reach the goal platform than control mice during the acquisition training. Former WIN-treated mice swam significantly faster than vehicle-treated animals during **b** the acquisition training and **d** probe trial. In contrast, **e** escape latencies did not differ significantly current WIN-treated WT animals and control animals. Furthermore, current WIN treatment did alter swimming speed (**f**, **h**). WIN treatment did not alter spatial reference memory as **c** current and **g** former treated mice showed a significant preference for the target quadrant. Chance level is indicated by a dashed line. Two-way (**a**, **b**, **e**, **f**) and one-way (**c**, **g**) ANOVA followed by Bonferroni multiple comparisons and unpaired t-test (**d**, **h**); n = 13–15. Data presented as mean ± S.E.M; *p < 0.05, **p < 0.01, ***p < 0.001

Across the 5 days of acquisition training current WIN-treated mice showed a similar decrease in escape latencies as control animals (Fig. 4e; two-way repeated measures ANOVA, *treatment* current: F(1,28) = 2.001, p = 0.1682). In addition, swimming did not differ between WIN- and vehicle-treated mice in the current group (Fig. 4f; two-way repeated measures ANOVA, *treatment* current: F(1,28) = 1.765, p = 0.1947).

In contrast, former WIN-treated WT mice showed a slower escape latency over the 5 days of training compared to control WT mice (Fig. 4a; two-way repeated measures ANOVA, treatment former: F(1,24) = 9.998, p = 0.0042). Furthermore, former WIN-treated mice swam significantly faster than vehicle-treated mice in the acquisition training (Fig. 4b; two-way repeated measures ANOVA, treatment former*:* F(1,24) = 14.97, p = 0.0007).

Twenty-four hours after the last acquisition trial, a probe trial was performed to assess spatial reference memory. Both the vehicle- and WIN-treated mice of the current group displayed a significantly higher preference for the target quadrant, as indicated by the relative time spent in the different quadrants of the pool (Fig. 4g; one-way repeated measures ANOVA followed by Bonferroni multiple comparisons, current: vehicle: F(3,52) = 59.81, p < 0.001; Bonferroni for target quadrant vs. left, vs. right and vs. opposite quadrant: p < 0.001; WIN: F(3,44) = 8.5, p < 0.001, Bonferroni target quadrant vs. left and vs. right: p < 0.01; target quadrant vs. opposite quadrant: p < 0.001). The swimming speed of the current group revealed no differences between the groups (Fig. 4h; current: unpaired t-test, F(14,13) = 11.85, p = 0.6721).

Similarly, mice of the former WIN treatment group showed a significant preference for the target quadrant (Fig. 4c; one-way repeated measures ANOVA followed by Bonferroni multiple comparisons, former: vehicle: F(3,56) = 20.76, p < 0.001; Bonferroni for target quadrant vs. left, vs. right and vs. opposite quadrant: p < 0.001; WIN: F(3,44) = 15.0, p < 0.001, Bonferroni for target quadrant vs. left, vs. right and vs. opposite quadrant: p < 0.001). However, WIN-treated animals of the former group swam significantly faster than same-aged control animals (Fig. 4d; unpaired t-test, current: F(13,13) = 3.480, p = 0.0009).

### Search strategy analysis in acquisition training reveals spatial navigation deficits in WIN 55,212-2 treated mice

In addition, the search strategies of mice during the acquisition training and probe trial were analyzed. During the first day of acquisition training, former vehicle and WIN-treated WT animals used predominantly a ‘random search’ strategy (Fig. 4i, j; vehicle: 60%, WIN: 87%; chi-square, treatment former: Day 1: p = 0.0781). On the second day of training, vehicle-treated animals used significantly less ‘random search’ compared to WIN-treated animals (Chi-square, treatment former: Day 2: p = 0.0118). As training progressed, non-spatial search strategies decreased in both vehicle- and WIN-treated mice. However, vehicle-treated WT animals shifted more quickly to spatial strategies, as non-spatial strategies were almost absent by day 4 of acquisition training (15%). In contrast, WIN-treated mice continued to show predominantly a ‘random search’ strategy (Day 4: 41%, Day 5: 35%) until the last day of acquisition training (Chi-square, treatment former: Day 3: p < 0.001, Day 4: p < 0.001, Day 5: p < 0.001). During the probe trial, the search strategies used by former WIN-treated mice did not significantly differ from vehicle-treated animals (chi-square, treatment former: Probe Trial: p = 0.1516).

Current WIN- and vehicle-treated WT mice showed mainly ‘random search’ strategies (vehicle: 75%, WIN: 69%) during the first day of acquisition training and the overall applied search strategies did not differ between the treatments (Fig. 4k, l: Chi-square, treatment current: Day 1: p = 0.1841). Over the subsequent days, the fraction of ‘random search’ declined and all WT mice used spatial strategies nearly exclusively. However, the search strategies of vehicle-treated mice shifted more quickly to spatial strategies compared to WIN-treated current mice. During the second day of training WIN-treated mice still used predominantly ‘random search’ (53%) strategies (chi-square, treatment current: Day 2: p < 0.001). During the third and fourth day vehicle- and WIN-treated mice used mainly a mixture of spatial strategies with vehicle-treated animals using more ‘directed search’ and ‘direct path’ than WIN-treated mice (Chi-square, treatment current: Day 3: p < 0.001, Day 4: p = 0.0017). During the last day of acquisition training, both groups used mainly spatial strategies to locate the platform with vehicle-treated mice using predominantly ‘focal search’ (27%), ‘directed search’ (14%), and ‘direct path’ (20%) strategies. In contrast, for WIN-treated mice ‘indirect search’ became the most dominant search strategy on the last day of acquisition training (Chi-square, treatment current: Day 5: p < 0.001). During the probe trial, 31% of WIN-treated animals used a ‘random search’ strategy, whereas this strategy was barley used by vehicle-treated WT mice (chi-square, treatment current: Probe Trial: p = 0.0269).

### Prolonged WIN 55,212-2 does not alter anxiety behavior in the elevated plus maze

**Fig. 5:**
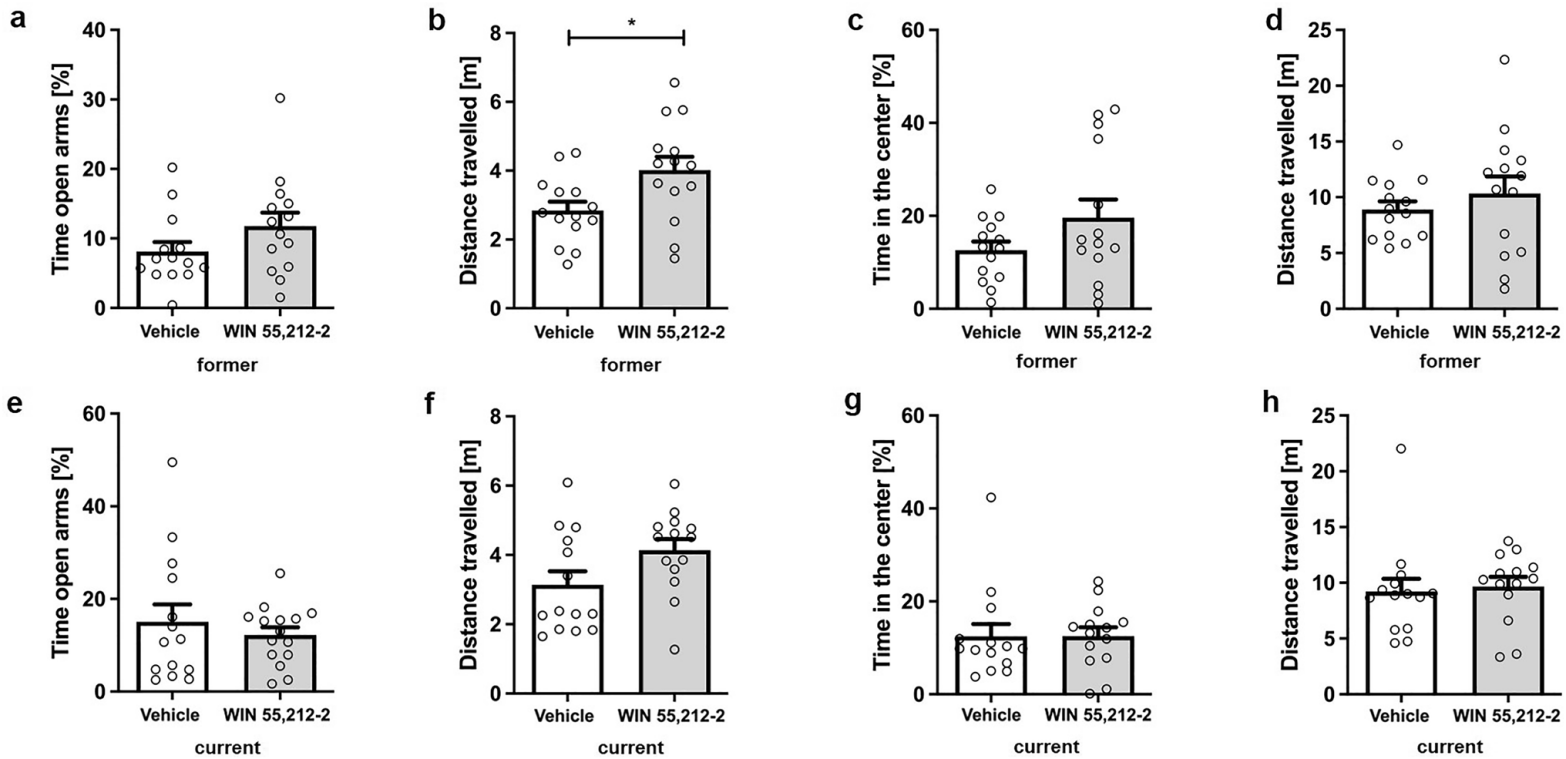
Effects of prolonged WIN 55,212-2-treatment on exploration and anxiety behavior in the elevated plus maze and open field. **a**, **e** Time in the open arms of the EPM was not influenced by WIN treatment, irrespective of treatment start. **b** Former WIN-treated mice traveled significantly further in the EPM than control mice. In contrast, **f** no significant differences in distance traveled was observed after current WIN treatment. Time in the center of the OF (**c**, **g**) did not differ between WIN-treated and control mice, irrespective of treatment start. Furthermore, WIN treatment did not influence distance traveled in the OF (**d**, **h**). Unpaired t-test, n = 14–15. Data presented as mean ± S.E.M; *p < 0.05

Former and current WIN-treated animals did not show a significant difference in the amount of time spent in the open arms compared to vehicle-treated controls (Fig. 5a, c; unpaired t-test, former: F(13,13) = 1.987 p = 0.1286, current: unpaired t-tests: F(13,13) = 5.816, p = 0.5713). However, former WIN-treated animals traveled significantly further than the control animals (Fig. 5b; unpaired t-test, former: F(13,13) = 2.311, p = 0.0191). In contrast, no significant difference in the distance traveled could be observed between current WIN- and vehicle-treated mice (Fig. 5d; unpaired t-test, current: F(13,13) = 1.457, p = 0.0586).

### Prolonged WIN 55,212-2 treatment does not alter anxiety behavior in the open field

Exploratory and spontaneous locomotor activity of mice was analyzed in the open field test. No significant difference was found between WIN-treated and control mice in terms of time spent in the center, regardless of treatment time (Fig. 5a, c; unpaired t-test, treatment: former: F(13,13) = 4.513, p = 0.1230; current: F(13,13) = 2.052, p = 0.9843). Furthermore, the distance traveled did not differ between WIN-treated animals and control mice (Fig. 5b,d; unpaired t-test, treatment: former: F(13,13) = 4.417, p = 0.3982; current: F(13,13) = 1.835, p = 0.7481).

### Prolonged WIN 55,212-2 treatment does not affect anxiety behavior in the dark light box

**Fig. 6:**
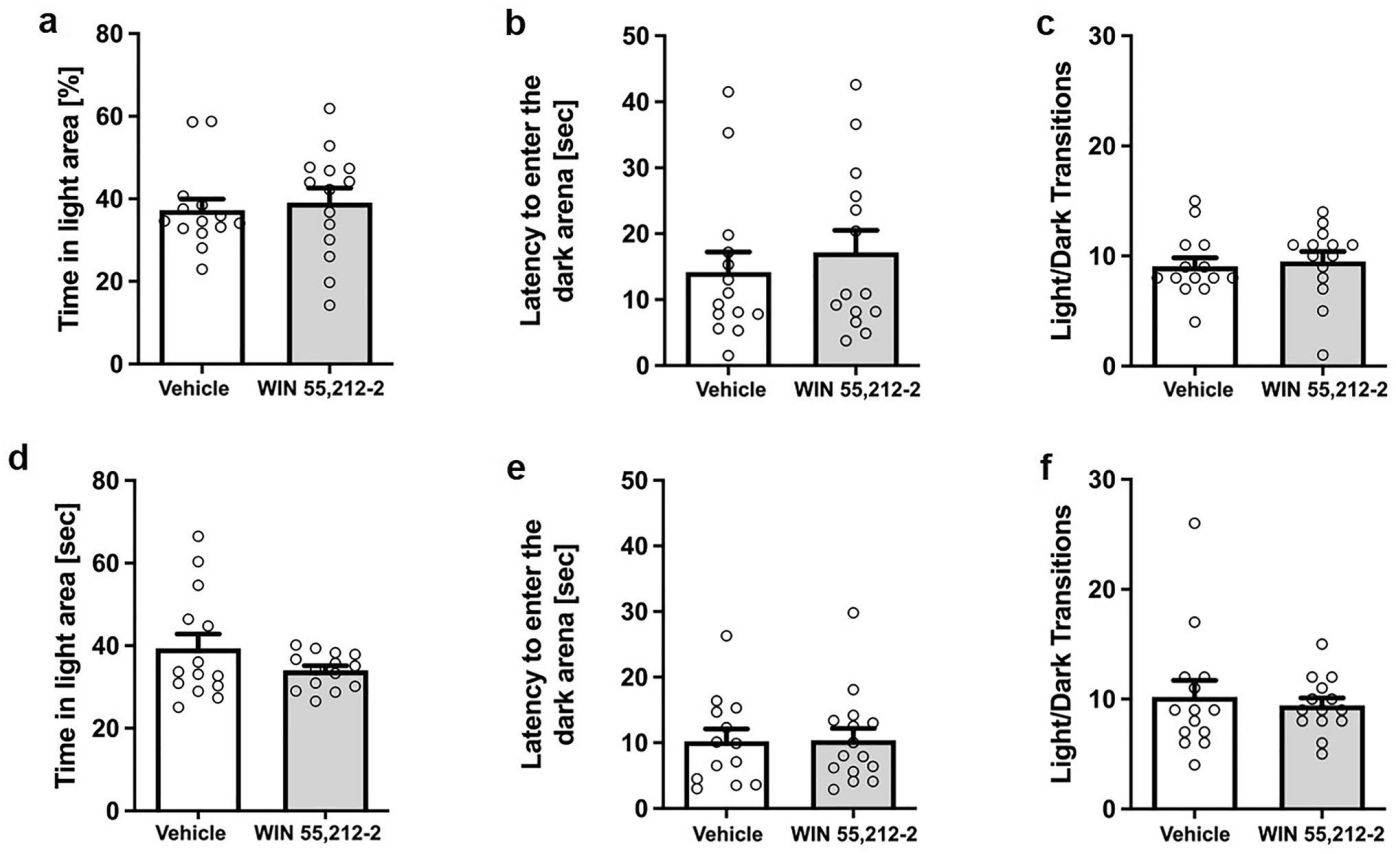
Effects of prolonged WIN 55,212-2-treatment on anxiety-related behavior in the Dark–Light Box. No significant difference in time spent in the light area in former (**a**) or current (**d**) WIN-treated and control mice. Furthermore, **b**, **e** the latency to enter the dark area did not differ between WIN- and vehicle-treated mice, irrespective of treatment start. **c**, **f** Number of light/dark transitions as a confounding factor of mobility in the dark light box did not differ between the treatment groups. Unpaired t-test, n = 14–15. Data presented as mean ± S.E.M

In both, the former and current treatment group, WIN-treated mice did not explore the light box longer than vehicle-treated mice (Fig. 6a, d; unpaired t-test, former: F(13,13) = 1.705, p = 0.3481; current: F(13,13) = 9.112, p = 0.1542). In addition, the latency to enter the dark area did not differ between treatment groups (Fig. 6b, e; unpaired t-test, former: F(13,13) = 1.199, p = 0.5125; current: unpaired t-test: F(13,13) = 1.063, p = 0.9493). In addition, the number of transitions between the light and dark box did not differ between the treatment groups mice (Fig. 6c, f; unpaired t-test, treatment: former: F(13,13) = 1.398, p = 0.7210; current: F(13,13) = 4.757, p = 0.6367).

### Prolonged WIN 55,212-2 treatment decreases cerebral glucose metabolism in several brain regions

To determine treatment effects on brain metabolism in vivo, former and current WIN-treated 7-months-old WT mice and age- and sex-matched control mice were scanned with ^18^F-FDG-PET. ^18^F-FDG distributed to the brain as well as to extracranial areas including the Harderian glands, myocardium, brown adipose tissue, intestines, kidneys, and the urinary bladder in all studied mice.

**Fig. 7:**
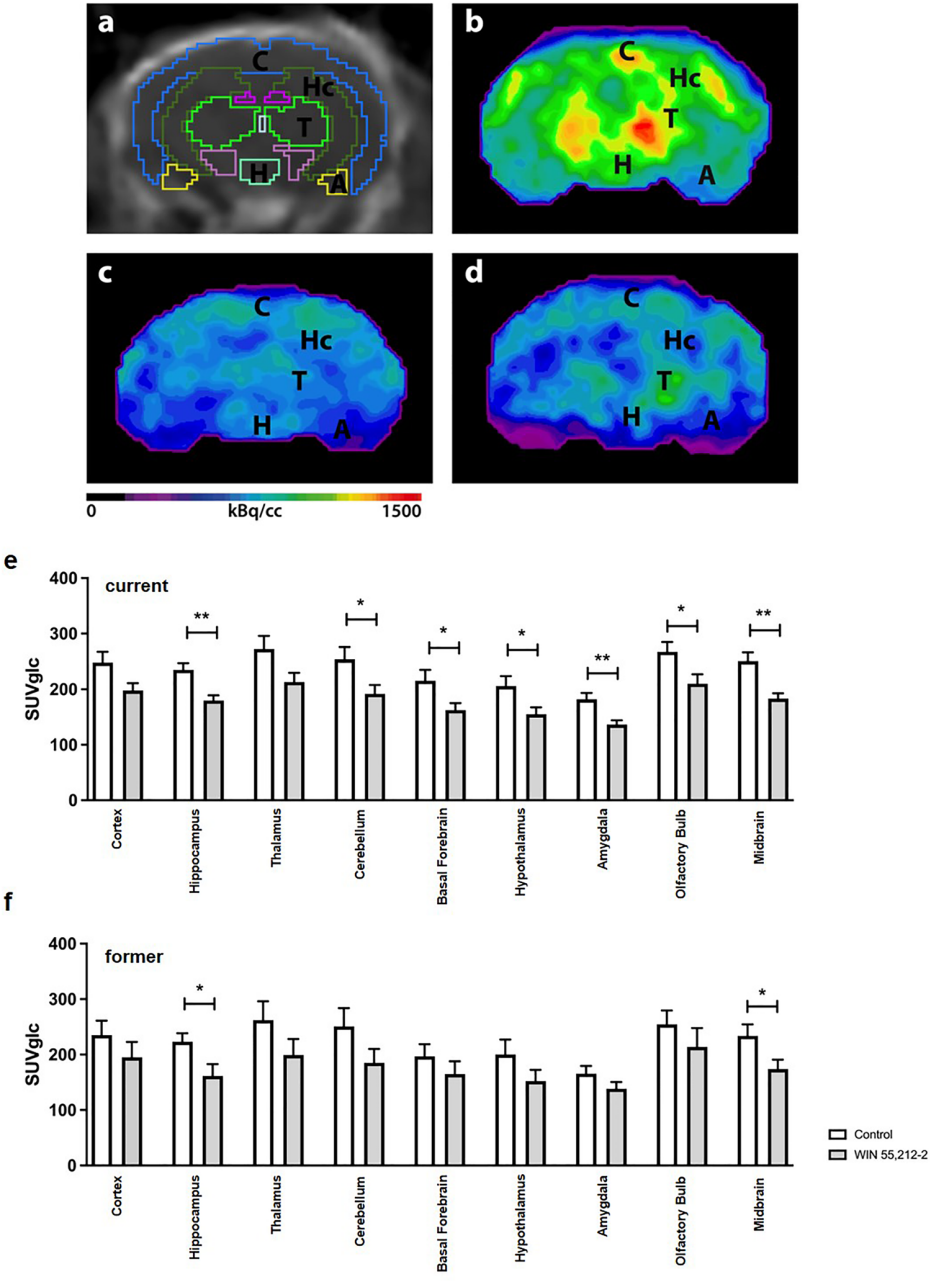
WIN 55,212-2 administration induces localized decreased cerebral glucose metabolism. **a** CT image in coronal view with predefined brain regions. ^18^F-FDG images of a representative **d** control WT mouse, **c** former WIN-treated, and **d** current WIN-treated WT mouse. Former **e** WIN-treated mice showed a hypometabolism in the hippocampus, cerebellum, basal forebrain, hypothalamus, amygdala, olfactory bulb and midbrain. **f** Current WIN-treated animals showed significantly lower SUV_Glc_ in the hippocampus and midbrain. Unpaired t-test; **p < 0.01, *p < 0.05 data presented as mean ± SEM

Former WIN-treated mice showed significantly lower ^18^F-FDG uptake in the hippocampus, amygdala, and midbrain regions compared to untreated mice (Fig. 7c, e; unpaired t-test, treatment: former: hippocampus: p = 0.0073; amygdala: p = 0.0254; midbrain: p = 0.0089).

Current WIN-treated mice also showed significantly lower ^18^F-FDG uptake in the hippocampus and midbrain regions compared to untreated mice (Fig. 7d,f; unpaired t-test, treatment: current: hippocampus: p = 0.0306; midbrain: p = 0.0384). All other regions did not show significant differences between current WIN-treated and control mice (unpaired t-test, treatment: current: cortex: p = 0.321; thalamus: p = 0.1963; cerebellum: p = 0.15; amygdala: p = 0.1567; forebrain: p = 0.3464; hypothalamus: p = 0.193; olfactory bulb: p = 0.3619).

## Discussion

Despite the promising potential of cannabis as a therapeutic for a wide range of conditions, long-term, regular cannabis use has been associated with significant cognitive, psychological, and neurobiological damage (Reddy 2022; Khalsa et al. 2022; Rubinstein Levy et al. 2022; Yucel et al. 2008, 2016). However, there is very little data on whether these brain-related changes are permanent or can recover with abstinence.

Although occasional adult cannabis use may not cause health problems, previous studies have shown that regular use can lead to a range of negative health effects, including depression, schizophrenia, cannabis use disorders, and poorer cognitive function (Feingold and Weinstein 2021; Shrivastava et al. 2011; Patel et al. 2020; Connor et al. 2021). In particular, cannabis use in adolescence has been linked to later depression, anxiety and impaired social functioning as well as changes in brain development and structure (Volkow et al. 2014; Brook et al. 2013; Jacobus et al. 2014). Various studies indicate that the adolescent abuse of Δ-9-tetrahydrocannabinol (THC), the psychoactive component of cannabis, increases the risk of long-lasting neurobiological changes in the adult brain (Fischer et al. 2020; Hoch et al. 2015). However, the risks of prolonged cannabis use during adulthood and the impact of different synthetic cannabinoids on the adult brain are less well studied.

Here we investigated the influence of prolonged WIN 55,212-2 treatment on healthy mature adult C57BL/6 J wildtype mice and its consequences on cerebral brain metabolism and behavior. WIN 55,212-2 is a synthetic highly potent cannabinoid receptor agonist that is a more potent CB1/CB2 agonist than THC, similar to many synthetic cannabinoids referred to as K2 and Spice drugs (Pintori et al. 2017; Yeruva et al. 2019). Noteworthy, it has been described that chronic synthetic cannabinoid users perform worse in working and long-term memory tests than non-users and recreational cannabis users (Livny et al. 2018; Cohen et al. 2017, 2020). Furthermore, Livny et al. (2018) demonstrated that chronic synthetic cannabis use lead to reduced total gray matter volume and impairments in the neural brain mechanisms responsible for working memory.

In the current study, mice were divided into two treatment groups to study the acute effects of WIN 55,212-2 treatment (current group) as well the effects of WIN 55,212-2 treatment after an extended washout phase (former group). All animals used in the study were healthy adult mice with an age roughly equivalent to that of 20–30 year old human (Dutta and Sengupta 2016; Wang et al. 2020). We demonstrated that long-term treatment with the synthetic cannabinoid in heathy adult WT mice resulted in recognition and spatial reference memory deficits without affecting anxiety-behavior. Furthermore, prolonged WIN 55,212-2 treatment induced hypometabolism in several brain regions.

Next to the assessment of therapeutic effects on behavior and cognition, the use of molecular imaging biomarkers allows therapy monitoring of molecular changes in-vivo. ^18^F-fluorodeoxyglucose positron emission tomography (^18^F-FDG-PET) is an established biomarker in Alzheimer’s dementia and other neurodegenerative diseases allowing a non-invasive assessment of cerebral glucose metabolism that reflects neuronal dysfunction. The reduction in brain glucose uptake highly correlates with cognitive deficits (Moodley and Chan 2014; Frisch et al. 2013). Recent results on ^18^F-FDG-PET imaging in different mouse models of Alzheimer’s disease and in WT mice showed its suitability in preclinical studies emphasizing a broader use in therapy studies (Bouter et al. 2018, 2021; Franke et al. 2020; Coleman et al. 2017; Macdonald et al. 2014).

However, the effects of WIN 55,212-2 treatment on cerebral glucose metabolism in healthy mice has not yet been studied in detail. Here we could demonstrate that WIN 55,212-2 treatment in early adulthood leads to a hypometabolism in several brain regions including the hippocampus, cerebellum, amygdala and midbrain, even after prolonged abstinence. Furthermore, prolonged acute WIN 55,212-2 treatment in 6-months-old mice reduced the glucose metabolism measured in the hippocampus and midbrain. To our knowledge, only one other study analyzed the effects of WIN 55,212-2 on the glucose metabolism using ^18^F-FDG PET: Martin-Moreno and colleagues focused on the cortex and hippocampus and demonstrated a reduced glucose uptake in both regions in 11-month-old WT mice after a 4-month long WIN exposure. Interestingly, WIN 55,212-2 did not influence the observed hypometabolism in Tg APP Alzheimer mice (Martin-Moreno et al. 2012). In line with our findings, previous autoradiography studies showed altered glucose metabolism after WIN 55,212-2 treatment. Pontieri et al. revealed a decreased glucose metabolism in the hippocampus and thalamus in male rats after a single low dose of 0.3 mg/kg WIN 55,212-2 (Pontieri et al. 1999). In addition, chronic, intermittent treatment with 1 mg/kg WIN 55,212-2 induced a hypometabolism in the hippocampus and thalamus with a contrasting hypermetabolism in the globus pallidus in adult WT mice (Mouro et al. 2018).

Neuroimaging studies in cannabis users showed a decreased brain metabolic activity in several brain regions after acute cannabis expose as well as after short periods of abstinence (Block et al. 2000; Lundqvist et al. 2001; Eldreth et al. 2004; Sevy et al. 2008; Cupo et al. 2021). However, the cerebral metabolism pattern after long periods of abstinence has not been well studied. Here we were able to demonstrate that the consumption of WIN 55,212-2 leads to persistent metabolic changes in the brain long beyond the period of intoxication. Strikingly, the effects on the observed hypometabolism were even more pronounced in the former group with a prolonged withdrawal period. In line with our findings, Sevy et al. (2008) observed a reduced glucose metabolism in young adults with a cannabis dependence in early full remission in the orbitofrontal cortex, putamen, and precuneus after 12 weeks of abstinence. Interestingly, it has been shown that glucose uptake in the brain of cannabis users is often highly similar to individuals with schizophrenia (Parkar et al. 2011).

CB1 receptors are widely expressed in the brain and regions with the highest densities of CB1 receptors include the hippocampus, cerebellum and basal forebrain. Therefore, the decreased glucose uptake after chronic WIN 55,212-2 treatment may be due to downregulation of CB1 receptors, which are localized in inhibitory interneurons and therefore inhibit the release of neurotransmitters such as glutamate, dopamine, GABA, and serotonin (Iversen 2003; Chang and Chronicle 2007). It could previously been shown that current cannabis users show an approximately 20% reduced CB1 receptor density on the brain that correlates negatively with the years of use (Hirvonen et al. 2012; Batalla et al. 2013). Interestingly, in rodents it could be demonstrated that adolescent animals display slower desensitization of CB1 receptors after chronic WIN 55,212-2 treatment than adults (Abush and Akirav 2010). However, the downregulation seems to be region specific and reversible. Hirvonen et al. (2012) demonstrated that the CB1 receptor density in chronic daily cannabis smokers returned to normal levels throughout the brain, with the exception of the hippocampus, after 4 weeks of abstinence (Hirvonen et al. 2012).

Our FDG-PET findings demonstrate that WIN 55,212-2 exposure leads to persistent metabolic changes in brain regions responsible for cognitive functions and memory. Thus, WIN treatment caused a hypometabolism in the hippocampus and midbrain in former and current treated adult WT mice. The hippocampus is crucially involved in learning and memory via its trisynaptic circuit. Phytocannabinoid and synthetic cannabinoids can interfere with hippocampal inhibitory synapses, leading to impaired cognition and memory (Tomas-Roig et al. 2017; Wilson and Nicoll 2002). Here we could demonstrate that prolonged WIN 55,212-2 treatment caused severe cognition memory and spatial reference memory deficits. In line with the present findings, long-term THC and WIN 55,212-2 administration has previously been described to cause persistent memory impairments in adolescent and adult rodents (Tomas-Roig et al. 2017; Abush and Akirav 2010; Bilkei-Gorzo et al. 2017). However, most studies analyzed the effects of cannabinoids on cognition during intoxication or a short washout period (Gorey et al. 2019).

Consistent with the observed hypometabolism, we found that cognitive deficits in WT mice were more pronounced in mice after a prolonged WIN 55,212-2 abstinence. Former WIN-treated animals showed severe memory deficits in the acquisition training of the MWM and the NOR. Importantly, the decreased glucose metabolism after more than 10 weeks of abstinence suggests that the observed memory deficits are not due to a residual effect of WIN 55,212-2 or an acute withdrawal effect. The fact that we detected differences in the acquisition training, but not in the probe trial, supports a specific effect of the drug on memory retrieval, as previously described (Wilson and Nicoll 2002; Tomas-Roig et al. 2017). In contrast, after 21 days of THC administration followed by a 28-day washout period Cha et al. (2006) did not detect any spatial learning deficits in adult Sprague Dawley rats in the MWM (Cha et al. 2006). Similarly, Gleason et al. (2012) described that a 10-day WIN 55,212-2 treatment in adolescent followed by a washout period resulted in fear conditioning deficits in adulthood, whereas treatment in adulthood did not cause long-term behavioral deficits (Gleason et al. 2012). These differences can likely be explained by the relatively short treatment periods in the previous studies.

Adult WIN-treated WT mice, irrespectively of treatment start, showed recognition memory deficits in in the NOR as they were not able to distinguish between a new and familiar object. Unlike other memory tests that can be clearly attributed to a specific brain region, the object recognition test appears to rely on several brain regions and neurotransmitter systems, including the hippocampus and perirhinal regions, making it especially difficult to interpret in terms of the underlying neurobiology (Balderas et al. 2013; Dere et al. 2007; Lueptow 2017; Warburton and Brown 2010).

In contrast to the observed memory deficits in the NOR, current WIN-treated mice showed a normal spatial memory performance in the MWM with respect to escape latencies and probe trial performance. However, a detailed analysis of the swimming strategies demonstrated allocentric-specific memory deficits in current WIN-treated mice. WIN-treated mice held on to non-spatial strategies longer than control animals during the acquisition training showing slight spatial navigation deficits. These deficits did not result in altered escape latencies as the non-spatial search strategies employed by WIN-treated mice were suffice to find the platform, but it nonetheless represents a behavioral phenotype. In line with these findings, Acheson et al. (2011) did not detect spatial memory deficits in adult Sprague Dawley rats after acute WIN 55,212-2 intoxication. However, adolescent WIN 55,212-2 treatment modulated swimming behavior in the MWM by decreasing thigmotaxis behavior (Acheson et al. 2011).

WIN 55,212-2 exposure did not modulate anxiety behavior in WT mice in the former or current group. Previous studies have produced conflicting results, with some showing antianxiety effects and others anxiolytic effects after WIN 55,212-2 or THC treatment (Kasten et al. 2017; Cassar et al. 2022). Similar to our results, Mouro et al. (2018) demonstrated that chronic, intermitted WIN 55,212-2 admiration during adolescence did not affect anxiety-like behavior in WT mice (Mouro et al. 2018). In addition, O'Shea et al. (2004) did detect increased anxiety in adolescent but not in adult rats after prolonged cannabinoid treatment (O'Shea et al. 2004). Furthermore, Onaivi et al. (1990) found no effects in mice but an anxiety-inducing effect in rats, when animals were treated with THC in adulthood (Onaivi et al. 1990). In contrast, O'Tuathaigh et al. (2010), Cadoni et al. (2008) and Schramm-Sapyta et al. (2007), demonstrated anxiolytic effects in adolescent and/or adult rats after prolonged THC administration (Cadoni et al. 2008; O'Tuathaigh et al. 2010; Schramm-Sapyta et al. 2007). In part, this inconsistency may be due to differences in strain or genotype sensitivity to THC or WIN 55,212-2 or the used dosage. Interestingly, chronic synthetic cannabis use in humans has been associated with increased depression and anxiety symptoms as well as schizotypal traits (Cohen et al. 2020).

Strikingly, former WIN 55,212-2 treated mice showed an increased locomotor activity and swimming speed. Similarly, Schramm-Sapyta et al. (2007) detected a modest locomotor-increasing effect in adolescent rats after THC treatment. However, they described a locomotor-decreasing effect in adult rats. Although locomotor activity may be a confounding factor in determining anxiety or memory performance, it is unlikely that these effects played a role in the observed memory deficits in former treated animals.

Taken together, our data demonstrate that long-term treatment with the synthetic cannabinoid WIN 55,212-2 in adulthood leads to a severe hypometabolism in the brain, even after a long period of abstinence. In addition, we have shown that WIN 55,212-2 exposure causes persistent memory deficits, especially when mice were treated in early adulthood. Our findings highlight the risks of prolonged WIN 55,212-2 use and underline the need for additional basic and clinical research before cannabinoids should be approved for clinical use.

## Data Availability

The raw data supporting the conclusions of this article will be made available by the authors, without undue reservation.
